# Effect of Olive-Pine Bottom Ash on Properties of Geopolymers Based on Metakaolin

**DOI:** 10.3390/ma13040901

**Published:** 2020-02-18

**Authors:** Eduardo Bonet-Martínez, Pedro García-Cobo, Luis Pérez-Villarejo, Eulogio Castro, Dolores Eliche-Quesada

**Affiliations:** 1 Department of Chemical, Environmental and Materials Engineering Higher Polytechnic School of Jaén, University of Jaen, Campus Las Lagunillas s/n, 23071 Jaén, Spain; ebonet@ujaen.es (E.B.-M.); pgc00002@red.ujaen.es (P.G.-C.); ecastro@ujaen.es (E.C.); 2 Department of Chemical, Environmental, and Materials Engineering, Higher Polytechnic School of Linares, University of Jaen, Campus Científico-Tecnológico, Cinturón Sur s/n, 23700 Linares (Jaén), Spain; lperezvi@ujaen.es; 3 Center for Advanced Studies in Energy and Environment (CEAEMA) Universidad de Jaén, Campus Las Lagunillas, s/n, 23071 Jaén, Spain

**Keywords:** geopolymers, metakaolin, biomass bottom ash, mechanical properties

## Abstract

In this research, the feasibility of using bottom ashes generated by the combustion of biomass (olive pruning and pine pruning) as a source of aluminosilicates (OPBA) has been studied, replacing the metakaolin precursor (MK) in different proportions (0, 25, 50, 75, and 100 wt.% substitution) for the synthesis of geopolymers. As alkaline activator an 8 M NaOH solution and a Na_2_SiO_3_ have been used. The geopolymers were cured 24 h in a climatic chamber at 60 °C in a water-saturated atmosphere, subsequently demoulded and cured at room temperature for 28 days. The results indicated that the incorporation of OPBA waste, which have 19.7 wt.% of Ca, modifies the characteristics of the products formed after alkaline activation. In general terms, the incorporation of increasing amounts of calcium-rich ashes results in geopolymers with higher bulk density. The compressive strength increases with the addition of up to 50 wt.% of OPBA with respect to the control geopolymers, contributing the composition of the residue to the acquisition of better mechanical behavior. The results indicate the potential use of these OPBA waste as raw material to produce unconventional cements with 28-day curing strengths greater than 10 MPa, and thermal conductivities less than 0.35 W/mK.

## 1. Introduction

Presently, one of the main tasks that the scientific-technical community must address in the field of materials is to solve the energy and environmental problems caused by the massive production of greenhouse gases, as well as to find new ways of use of waste deposited in landfills. Everyone is aware of the need to incorporate materials, whose manufacturing or processing does not involve the emission of large amounts of CO_2_ into the atmosphere.

For this reason, the study and development of new cementitious materials alternative to Portland cement is a priority research line of great interest worldwide. The development of these new materials aims to minimize pollutant emissions into the atmosphere, as well as, significant energy savings.

The cement industry is a highly polluting industry. One of the major problems that the cement industry presents is that during the manufacturing process, a large amount of gaseous and solid emissions such as greenhouse gases (CO_2_), other polluting gases such as NO_x_, SO_2_, and NH_3_ are generated, as well as dust. In addition, the cement manufacture implies an intense exploitation of natural resources (quarries) [1]. It is estimated that between 6% and 8% of CO_2_ emissions emitted into the atmosphere have an origin in the cement industry [2]. This industry emits between 0.7 and 1.2 tons of CO_2_ per ton of clinker manufactured [2] and needs to use energy intensively during its production process. The heat required for the manufacture of the Ordinary Portland Cement (OPC) is 3100–3600 kJ/kg [3]. Thus, only the decomposition of calcite in Portland cement production generates 0.54 tons of CO_2_, in addition to almost a ton of carbon dioxide released during the process, especially in the grinding and clinkerization stages [4]. In summary, the production of cement clinker involves the expenditure of a large amount of energy during its production, almost 810 kg of carbon dioxide (CO_2_), 1 kg of sulfur dioxide (SO_2_) and 2 kg are discharged into the atmosphere of nitrogen oxides (NO_x_) per ton of cement produced [5,6,7]. Taking into account that cement production is approximately 3700–4000 million tons per year, this implies a substantial CO_2_ footprint. Consequently, the reduction of limestone in the raw material and, therefore, the change in its chemistry and/or the clinkerization temperature, could lead to lower CO_2_ emissions.

For all the above, the cement industry faces a series of challenges for the future, such as a reduction in energy consumption and the reduction of fossil fuel consumption. Success in both of these challenges would lead to a reduction in CO_2_ emissions. Currently, the use of alternative raw materials that contain CaO, such as class C fly ash or blast furnace slag, is contributing to the reduction of energy consumption. The saving of fossil fuels is achieved by using other alternative fuels such as used tires, sewage sludge and paper production wastes. In addition, another challenge of this sector is the improvement of the efficiency of the process, which would mean the implementation of technological improvements.

With this background, it is necessary to develop new alternative materials to Portland cement, in whose manufacture no polluting gases are emitted and significant energy savings are obtained. Geopolymers or materials obtained by alkaline activation of aluminosilicates as natural products (clays) or industrial by-products (furnace slag and/or fly ash) are among the most promising alternatives. The alkaline activators used are strong alkaline solutions (NaOH, Na_2_CO_3_ or hydrated alkali silicates). These alkaline materials can lead to a reduction in CO_2_ emissions of between 55%–75% compared to the manufacture of Portland cement [8]. Geopolymers are characterized by having low hydration heats, high mechanical performance, and good durability against different aggressive agents (acidic media [9], sulfate attack [10], fire [11], etc.).

Geopolymers are binders basically formed by two components: a dusty material of aluminosilicate nature that is the precursor, and an alkaline activator. The reaction processes that are carried out during the alkaline activation must be considered to be a set of complex transformations of the starting solid that in its final state lead to a condensed structure with cementing properties [12,13]. These alkali-activated materials (AAM) or geopolymers, in a context of environmental degradation and due to the emphasis and widespread demand for sustainability, have increasingly attracted the attention of researchers. The growing interest in these new materials is because they can be obtained from different precursors rich in Si-Al minerals, including waste and industrial by-products, through a low temperature manufacturing process, which implies great environmental benefits compared to traditional cementing materials. Among the industrial waste most commonly used as precursors of geopolymers can be found fly ash or metal slag. A source of aluminosilicates widely studied as a raw material in the manufacture of geopolymers with satisfactory results has been coal fly ash. However, the use of ashes from biomass sources as raw material or alkaline activator in a synthesis of geopolymers aroused great interest among scientists. This is due to the fact that most of thermal power generation plants will be progressively closed, as a result, of fossil fuel consumption reduction policies that reduce or prevent climate change. These plants are replaced by plants that use biomass ash as a main fuel. Thus, it is expected that by the year 2050, between one third and one half of the world’s primary energy consumption will have a biomass origin [14].

The difference between coal and biomass ashes is the composition. Coal fly ashes consist mainly of iron, aluminum, and silica [15], interesting elements to be used as raw material in geopolymers, since sources of silica and alumina are needed. In addition, they usually have a fairly important fraction of amorphous material, which makes them quite chemically reactive. Biomass ashes are mainly composed of silica and amorphous alumina, which is the most chemically reactive, which makes them an appropriate material to be used in the manufacture of geopolymers [16]. However biomass ashes have a higher degree of crystallinity, which makes them less reactive, especially calcium and potassium salts. 

Andalusia, a region located in southern Spain, has a large area dedicated to the cultivation of olive groves (1.4 million hectares). This sector generates a large number of by-products such as olive stones or pits, olive leaves, and olive pruning biomass. The biomass potential available in this region is 3,327 kton per year [17]. The main recovery of these biomass wastes is their use as fuel for electricity generation. Likewise, the forestry industry is another potential source of biomass. The combustion of biomass generates other wastes: fly and bottom ashes that are extracted from the different stages of the combustion process. These ashes as can be used as raw materials in the manufacture of geopolymers as an alternative to their deposition in landfills.

The olive biomass ashes have a high content of alkaline and alkaline earth salts, especially potassium, which makes them a priori excellent candidates to increase the pH, in the geopolymeric reaction and can substitute, if not totally, but partially, the classic alkaline activators used as sodium silicate or waterglass [18]. Thus, according to recent research [19], ashes from the olive industry do not exhibit pozzolanic activity due to their low silica and alumina content, which prevents them from being used as Portland cement active additive. These ashes have been used, however, as partial substitutes in ceramic materials [20] and mortars [21].

The objective of this work was to use the residue, biomass bottom ashes, from the combustion of olive and pine pruning, as a substitute for the metakaolin precursor to obtain alkali-activated materials, geopolymers. The replacement of metakaolin by the olive-pine pruning bottom ash (OPBA) in different quantities (0, 25, 50, 75, and 100 wt.%) was studied. The chemical, physical, mechanical, and thermal properties of the geopolymers obtained were analyzed. Therefore, the intention is to develop more sustainable cementitious materials, alternative to Portland cement, which depend on a smaller scale of natural resources and include recycled materials that can become more economic and with lower environmental impact than conventional ones.

## 2. Materials and Methods

### 2.1. Raw Materials

The raw materials used were metakaolin (MK), which was obtained after a calcination heat treatment at 750 °C of kaolin provided by the company Caobar S.A. located in Taracena (Guadalajara, Spain). The kaolin provided by this company had an adequate particle size, so it was not necessary to subject it to subsequent grinding treatment. Biomass bottom ashes were purchased from the Aldebarán Energía de Guadalquivir (Andújar, Jaén) and come from the calcination of biomass from the pruning of olive and pines trees. These ashes were provided with a quite heterogeneous particle size, so once received in the laboratory it was necessary to perform a grinding stage in a ball mill and finally another sieving stage until reaching a particle size of 0.150 mm.

As alkaline activator, a basic solution of 8 M sodium hydroxide was used; it was mixed with sodium silicate in aqueous medium, obtaining a pH value of 13. The sodium hydroxide was provided by Panreac SA, with a purity of 98 %. The sodium silicate solution was purchased from Panreac S.A., and has a density of 1365 kg/m^3^ and a pH of 11.5. The composition by weight of this commercial sodium silicate is: 29.2% SiO_2_; 8.9% Na_2_O and 61.9% H_2_O.

### 2.2. Raw Materials Characterization

A laser diffractometer Malvern Mastersizer 2000 was used to measure the particle size distribution of raw materials. This device analyzes the particle size distribution of between 0.02 and 1500 µm of any solid material dispersed in liquid medium using diffraction technology laser light.

Raw materials and geopolymers were evaluated by X-ray diffraction (XRD) to know their crystalline phases. An Empyrean X-ray diffraction equipment with a PIXcel-3D detector of PANalytical (Malvern, United Kingdom) was used, using Cu Kα radiation (λ = 1.5406 Å) at a voltage of 40 kV and an amperage of 40 mA, a range 2θ from 10° to 60°, and a step size of 0.02°. HighScore software (version 4.7 version, Malvern PaNalytical, Malvern, United Kingdom) was used for the identification of phases. The chemical analysis of the raw materials was carried out using the Philips Magix Pro model PW-2440 X-ray fluorescence equipment (XRF), a sequential dispersive wavelength spectrometer with a 4 kW X-ray generator.

The real density of the raw materials was measured by helium picnometry using an AccuPyc II 1340 equipment. The real density of MK and OPBA waste resulted in 2631 and 2564 kg/m^3^ respectively.

### 2.3. Preparation of MK-OPBA Geopolymers

The alkaline activating consists of a solution of sodium hydroxide and sodium silicate. First, an 8 M sodium hydroxide solution is prepared (80 g of NaOH are dissolved in 195 g of water), to which the appropriate amount of sodium silicate (300 g) is added continuously and slowly. The entire mixing process is carried out on a magnetic stirrer with an approximate duration of ten minutes. After stirring, the mixture is allowed to cool to room temperature. The alkaline activator solutions are finally subjected to a pH measurement. These pH measurements were made with a Crison Basic 20 pH meter. In all series, the activating solution has remained constant, as well as, the liquid / solid ratio = 0.85 which allows a workability/consistency suitable.

The formed geopolymers are prepared by replacing different weight ratios of MK precursor (0, 25, 50, 75, and 100 wt.%) by the olive-pine bottom ash residue. The control geopolymers contain only as a source of aluminosilicate, MK (0% OPBA). 

The necessary quantities of MK and the OPBA residue (Table 1) are weighed and mixed in a planetary kneader. The planetary kneader has two rotation speeds and two translation speeds. The rotation speed of the blade on its axis: slow: 140 ± 5 min^−1^ and fast: 285 ± 10 min^−1^; and the translation speed around the center of the container, slow: 62.5 ± 5 min^−1^ and fast: 125 ± 10 min^−1^.

The raw materials were mixed for five minutes in solid state until a homogeneous solid mixture is achieved. After this time, the activator solution was added slowly, reaching a time of pouring and mixing slowly of two minutes. Then the kneading was done at fast speed mixing for another ten minutes, thus achieving 17 minutes of mixing for each series of geopolymers.

**Figure 1:**
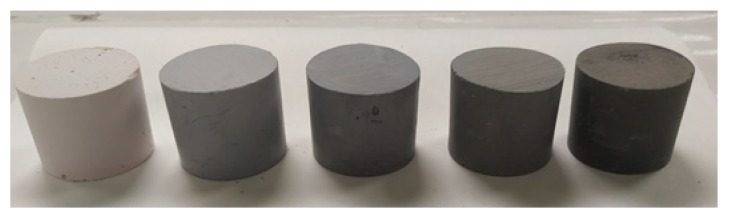
The geopolymers obtained, from left to right: 0 wt.% OPBA, 25 wt.% OPBA, 50 wt.% OPBA, 75 wt.% OPBA and 100 wt.% OPBA.

Once the kneading was carried out, the geopolymeric mixture is poured onto polyethylene containers, slowly and providing small blows to achieve a better compaction of the geopolymer in the mold. The containers were covered with a plastic film, thus ensuring that the amount of water lost in the geopolymer mixture is the minimum, maintaining the humidity conditions while the material set and hardens to prevent rapid settling and/or carbonation processes. Subsequently, the molds were transferred to the climatic chamber where they are cured at 60 °C with a water-saturated atmosphere for 24 h. The geopolymer precursors were then demoulded and cured at room temperature (22 °C and relative humidity of 57%) for 28 days. Specimens with a diameter of 35 mm and 50 mm height were used for physical and mechanical tests (Figure 1). 

The geopolymers were designated as xMK-yOPBA where *x* denotes the MK content (wt.%) and y the OPBA wt.% content. It can be seen in Table 1 that as the amount of substitution of MK by OPBA increases, the Si / Al molar ratio increased, while the Na / Si molar ratio remained practically constant.

### 2.4. Characterization of MK-OPBA Geopolymers

The degree of reaction was determined by attacking 1 gram of geopolymer (ground and subsequently sieved to a particle size of 150 μm) with a solution of HCl (1:20). Then, the solid was filtered under vacuum, dried in an oven for 1 or 2 hours and then calcined at 1000 °C for 3 h. The aluminosilicate gel formed was dissolved in the HCl, while the fraction of ash that does not react remains in the insoluble residue. The degree of reaction is calculated by the loss of mass produced during the attack of the acid to the geopolymer, according to the Equation (1):

Degree of reaction (%) = (1 − calcined mass) × 100(1)

The bulk density determination was carried out according to the UNE-EN 772-13, 2001 Standard [22]. Water absorption was determined according to ASTM C373 standard [23]. 

Mechanical properties of geopolymers were conducted by measuring compressive strength. This assay was carried out according to the standard procedure UNE-EN-772-1, 2011 [24] using a Material Test System (MTS) 810 Material Testing Systems laboratory press. The geopolymers were tested by applying a constant displacement rate of a 2 mm/min.

Thermal conductivity of the alkali-activated materials was determined at 10 °C using a Heat Flow Meter (FOX 50, TA Instruments, New Castle, DE, USA) in accordance with ISO 8302, 1991 [25]. A 35 mm of diameter and 15 mm of height specimens were used to determine thermal properties.

An equipment of attenuated total reflectance-Fourier Transform Infrared spectroscopy (ATR-FTIR, Vertex 70, Bruker, Billerica, Massachusetts, United States) was used to characterize the geopolymers.

Microstructure of fractured surfaces were examined by means of scanning electron microscopy (SEM) using a SM 840 model, JEOL equipment, Akishima, Tokio, Japan, and assisted by Energy dispersive X-ray Spectroscopy (EDS). The working distance was 8.5 mm, the acceleration voltage was 20 KV and beam current was 700 pA. Samples were placed on an aluminum grate and coated with carbon using the JEOL JFC 1100 sputter coater.

## 3. Results and Discussion

### 3.1. Raw Materials Characterization Results

Table 2 shows the chemical composition of raw materials, the MK and the OPBA waste determined by XRF. The MK is composed almost entirely of silica (58.03%) and alumina (40.29%).

The OPBA waste have a high silica content (46.10%), at the same time presenting high content in calcium oxide (19.65%) and alumina (12.04%). Other oxides such as magnesium oxide (3.71%), potassium oxide (4.59%) or iron oxide (4.78%) are also significant. The loss on ignition (LOI) is of 5.58 wt.%. The sum of SiO_2_ and Al_2_O_3_ in the OPBA waste accounts for 58 wt.%. Therefore, OPBA waste is a good candidate for the substitution of MK in the synthesis of geopolymers.

**Figure 2:**
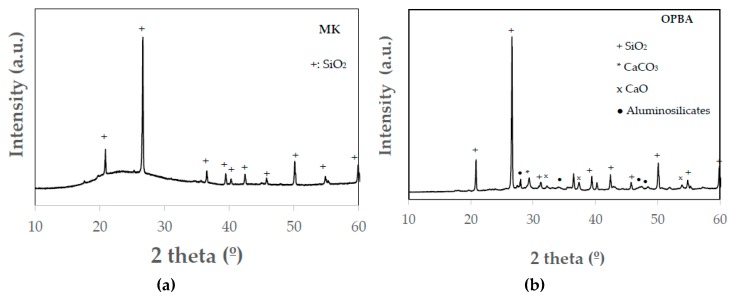
XRD patterns of raw materials: metakaolin: (**a**) Metakaolin (MK) and (**b**) olive-pine bottom ash (OPBA)**.**

The mineralogical composition of MK determined by XRD indicates that the precursor presents quartz as the only crystalline phase (Figure 2). The halo observed between 2 theta 20–30° is mainly attributed to the degree of amorphousness of the precursor. The OPBA waste diffraction pattern (Figure 2) indicates that biomass bottom ashes are mainly composed of silica and calcium carbonate. Also presenting a large number of small diffraction peaks corresponding to calcium oxide and some aluminosilicates, according to the XRF data (Table 2).

**Figure 3:**
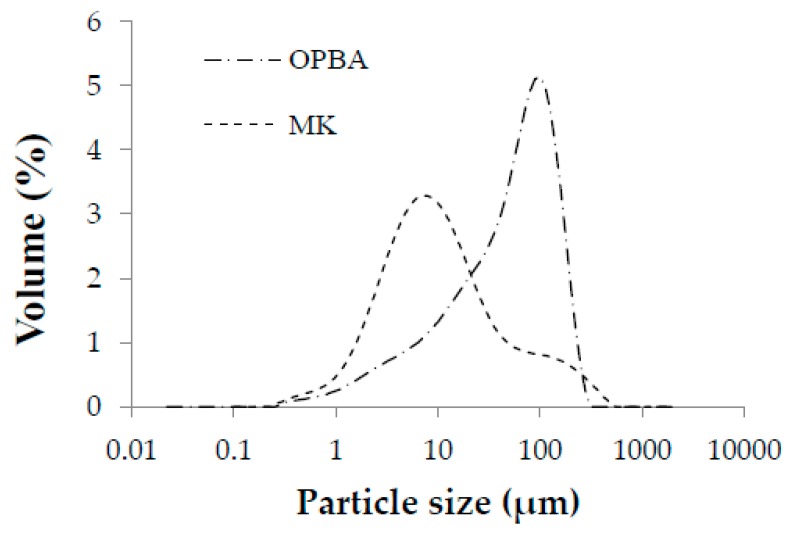
Particle size distribution of raw materials: metakaolin (MK) and olive-pine bottom ash (OPBA).

The particle size distribution of the precursors MK and OPBA is shown in Figure 3. The particles present in the MK precursor were thinner than those present in the OPBA waste. The average particle size D_50_ is 9.6 µm for the MK and 52.6 µm for the OPBA waste. The larger particle size of the OPBA indicates that the residue has a smaller surface area and therefore absorbs less water, giving a more fluid paste as larger amounts of OPBA precursor are incorporated.

The MK is made up of particles of a similar size to the silt (0.002–0.063 mm) (79.0%), containing to a lesser extent particles of clay size (<0.002 mm) (15.25%) and sand size (0.063–2 mm) (12.89%). However, the OPBA waste have higher percentage of sand-sized particles (51.5%), presenting a lower percentage of particles of silt size (33.3%) and clay size (15.25%). The particles present in the precursor MK are smaller than the OPBA waste particles.

**Figure 4:**
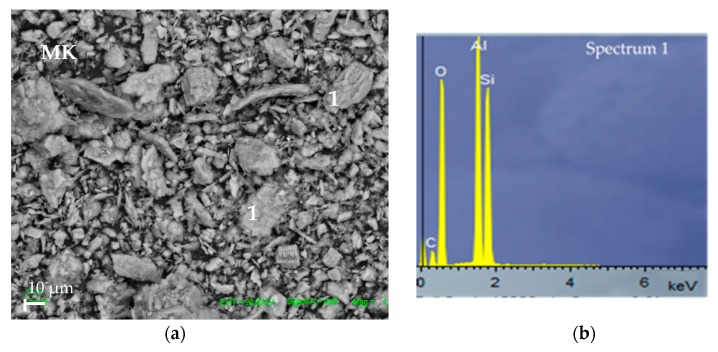

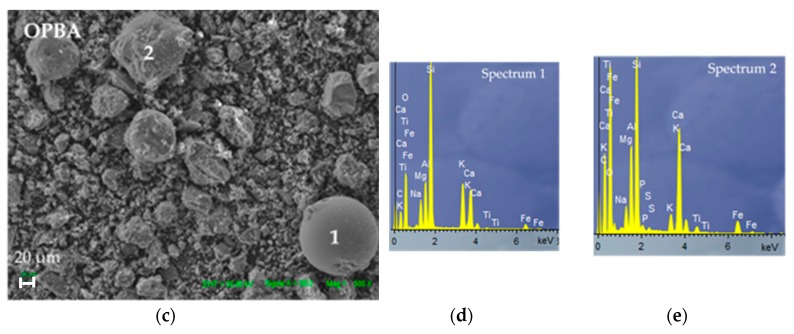
SEM of raw materials: (**a**,**b**) metakaolin (MK) and (**c**,**d**,**e**) olive-pine bottom ash (OPBA).

Micrographs obtained by Scanning Electron Microscopy (SEM) and EDS analysis corresponding to the raw materials MK precursor and the OPBA waste are shown in Figure 4.

The MK and OPBA have a heterogeneous structure with a widespread particle size distribution. The OPBA particles are larger than MK particles, according to the particle size distribution data. The MK particles have morphology of deformed flakes all rich in Si and Al, the main constituents of MK. The biomass bottom ashes present two types of particles, spherical and irregular particles. Both particles are rich in Si, Al, Ca, Mg and K, being the richest in calcium acicular particles.

### 3.2. MK-OPBA Geopolymers Characterization Results

#### 3.2.1. FTIR of MK-OPBA Geopolymers

**Figure 5:**
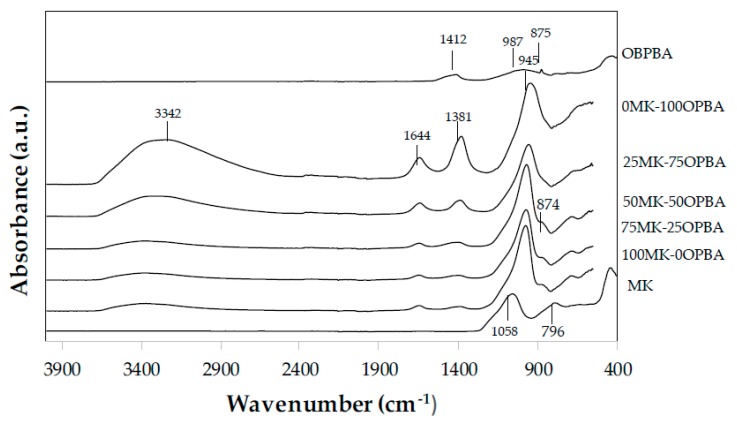
FTIR spectra of raw materials: MK and OBPBA and geopolymers xMK-yOPBA.

In the hardening process of the xMK-yOPBA geopolymers, a series of chemical bonds are formed as a consequence of the chemical reactions that occur once the activating solution is added to the mixture. These chemical bonds can be observed by FTIR. Figure 5 shows the FTIR spectra of the geopolymers after 28 days of curing. As a comparison, the FTIR spectra of the MK and OPBA raw materials are also shown. In the raw materials MK and OPBA had a band centered at 1058 cm^−1^ or 987 cm^−1^, respectively characteristic of the stretching modes of the T-O-T bond, where T can be Si or Al can be observed. This band, with the incorporation of the activating solution, shows a shift towards lower wavenumbers (974–945 cm^−1^) for geopolymers containing between 0–100 wt.% of OPBA waste. The displacement observed can be associated with the dissolution of the aluminosilicate source. This displacement indicates that the silicate group has geopolymerized forming the geopolymeric gel. The displacement of the band towards greater wavelengths as the percentage of OPBA decreases, could be influenced by a lower availability of calcium in the specimens. The presence of the N-A-S-H gel has a greater preponderance on the appearance of the second gel, (N, C)-A-S-H, whose formation is subsequent due to the incorporation of a greater amount of calcium [26]. The band that appears at 796 cm^−1^ on the MK precursor can be assigned to bending of the Si-O-Si bonds in the network of the unreacted silica [27]. In the region between 3800 and 1400 cm^−1^ two bands can be observed. The first very wide band centered between 3369–3342 cm^−1^ is attributed to the vibration of the –OH bond of the molecular water present freely or physically absorbed on the surface or pores of the gel [28]. The second band centered between 1650–1644 cm^−1^ corresponds to the vibration by deformation of the H-OH bond, since the high alkali content in the pore solution prevents evaporation of the water [29]. The greater intensity of these bands as increasing amounts of ash are incorporated as raw material, can be attributed to the greater number of water molecules present in the geopolymer as the SiO_2_/Al_2_O_3_ ratio increases [30], as well as to a lower degree of geopolymerization reaction for ash contents greater than 50 wt.% (Table 1), since water is consumed during the different reaction stages. In the region between 1400–900 cm^−1^, in addition to observing the bands centered at 974–945 cm^−1^ associated with the asymmetric stretching vibrations of the Si-O-Si and Si-O-Al bonds of the geopolymeric gel formed, another band centered between 1409–1381 cm^−1^ can be observed that corresponds to the asymmetric tension vibrations of the CO bonds indicating the presence of sodium carbonates (𝐶𝑂_3_^2−^). This bands is complemented by a shoulder band centered at 877–793 cm^−1^ that corresponds to the vibration by symmetric deformation of the O−C–O bonds. The bands present in the region between 800–600 cm^−1^ are related to the stretching vibration of the Al-O bonds, specifically for Al ions with coordination 4. Another band located at 880 cm^−1^ should be assigned to Si-O-M in the asymmetric stretching mode [28].

#### 3.2.2. XRD of MK-OPBA Geopolymer

**Figure 6:**
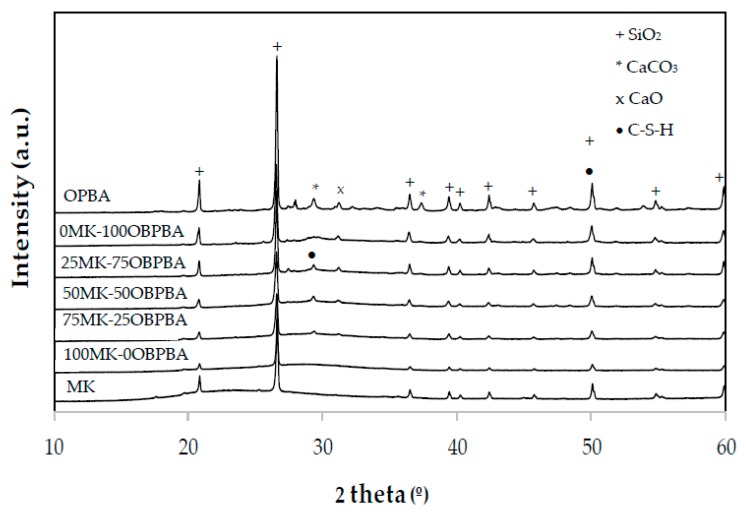
XRD patterns of raw materials MK and OPBA the geopolymers xMK-yOPBA.

**Figure 7:**
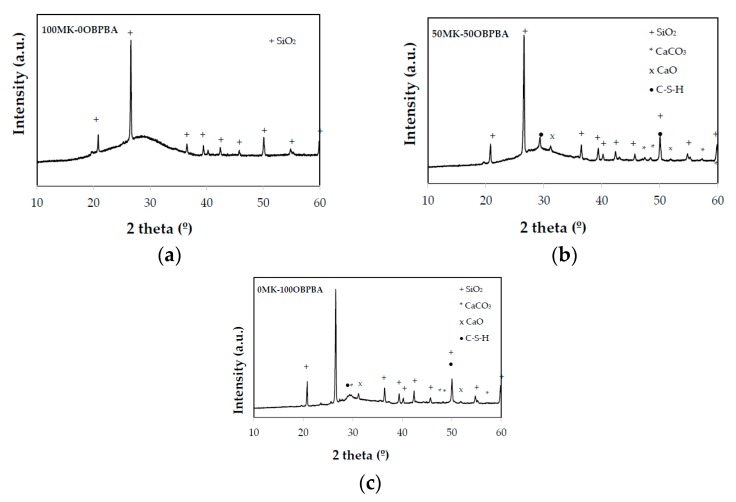
XRD patterns of (**a**) 100MK-0OPBA, (**b**) 50MK-50OPBA and (**c**) 0MK-100OPBA geopolymers.

Figure 6 shows the diffractograms of the synthesized geopolymers and, as a comparison, the diffractograms of the raw materials, MK and OPBA. The presence of a series of diffraction peaks present in the raw materials that indicates the presence of crystalline substances that are not involved in the geopolymerization reaction can be observed. These peaks are les intense, indicating that the surface of the crystals is attacked in the geopolymerization reaction due to the aggressive medium in which they are found. No new crystalline phases appearing in the geopolymerization process of 100 MK-0OPBA geopolymers. In geopolymers that use OPBA waste as raw material, the broad diffraction peaks corresponding to the precipitation of the C-S-H gel can also be observed [26]. The diffraction peaks of the quartz, present in both raw materials the MK and the OPBA, or the calcium carbonate and calcium oxide present in the OPBA are observed. The presence of amorphous substances can also be observed, due to the deviations observed in the baseline with respect to the raw material MK between the values of 2θ between 20° and 35° (Figure 7). This halo can be observed more accurately in Figure 7. This fact could indicate the appearance during the geopolymerization reaction of an amorphous geopolymeric gel as indicated FTIR data.

#### 3.2.3. Apparent Porosity, Water Absorption and Bulk Density of MK-OPBA Geopolymers

**Figure 8:**
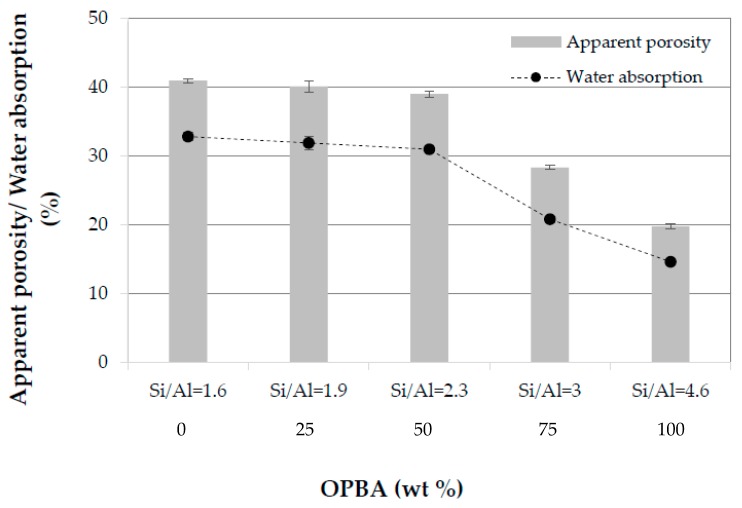
Apparent porosity and water absorption of the MK-OPBA geopolymers after 28 days of curing as function of the OPBA waste wt.% content and the Si/Al molar ratio.

The porosity of geopolymers to be used as building materials makes them vulnerable to weathering and chemical attack. Control geopolymers and specimens containing up to 50 wt.% of OPBA have low bulk density and high apparent porosities between 41%–39% for control samples (100MK-0OPBA) and 50MK-50OPBA geopolymers, respectively. The addition of larger amounts of bottom ash (75–100 wt.%) produced a more pronounced decrease in apparent porosity up to 20 % for geopolymers that use only OPBA waste (0MK-100OPBA) as raw material (Figure 8). This fact could be due to the interpenetrating action and the filling effect of biomass bottom ashes. The water absorption, an indirect measure of the open porosity of geopolymers, follows the same trend as the apparent porosity. Control and geopolymers containing up to 50 wt.% of OPBA waste have similar water absorption values, between 32.8% for 100MK-0OPBA and 31.0% for 50MK-50OPBA specimens. The incorporation of 75–100 wt.% of OPBA waste produced a more pronounced decrease to 14.7% for 0MK-100OPBA geopolymers (Figure 8).

**Figure 9:**
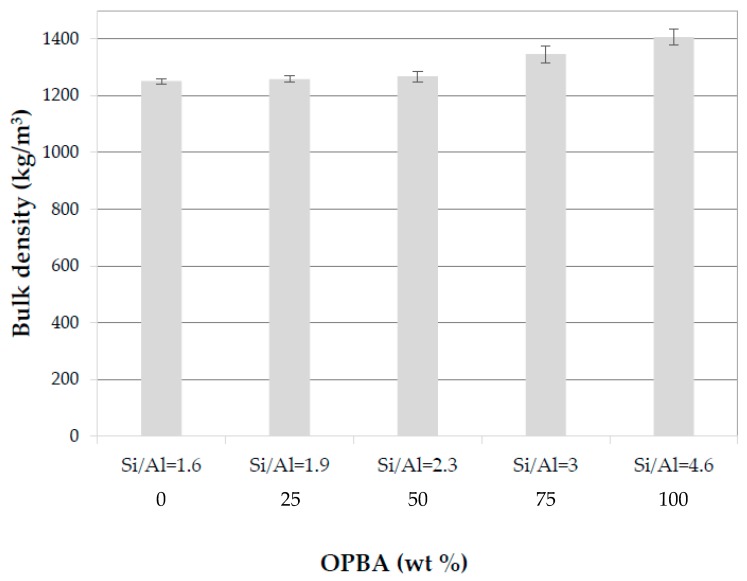
Bulk density of the MK-OPBA geopolymers after 28 days of curing as function of the OPBA waste content and the Si/Al molar ratio.

The data of bulk density of the different OPBA geopolymers after 28 days of curing are shown in Figure 9. The bulk density of the control geopolymer 100MK-0OPBA is 1251 kg/m^3^. The replacement of 25–50 wt.% of OPBA by MK produced a slight increase in bulk density, up to 1268 kg/m^3^ for the 50MK-50OPBA geopolymers. The addition of 75–100 wt.% of OPBA waste produced a remarkable change in the bulk density increasing up to 1407 kg/m^3^ for geopolymers containing only OPBA (0MK-100OPBA), according to apparent porosity data. The real density of the OPBA, 2546 kg/m^3^, is lower than the real density of the MK, 2631 kg/m^3^. Therefore, the increase in the bulk density and the decrease in apparent porosity of the MK-OPBA geopolymers may be due to the filling effect of the waste, which acts as microfillers. This results in an increase of the bulk density of the specimens as a denser microstructure is formed, mainly due to the different geopolymerization mechanism reactions caused by the calcium content of OPBA wastes. The addition of other complementary materials, such as silica fume, fly ash, and granulated blast furnace slags, produces the same filling effect, increasing the bulk density of the cement paste.

#### 3.2.4. SEM Study of MK-OPBA Geopolymers

**Figure 10:**
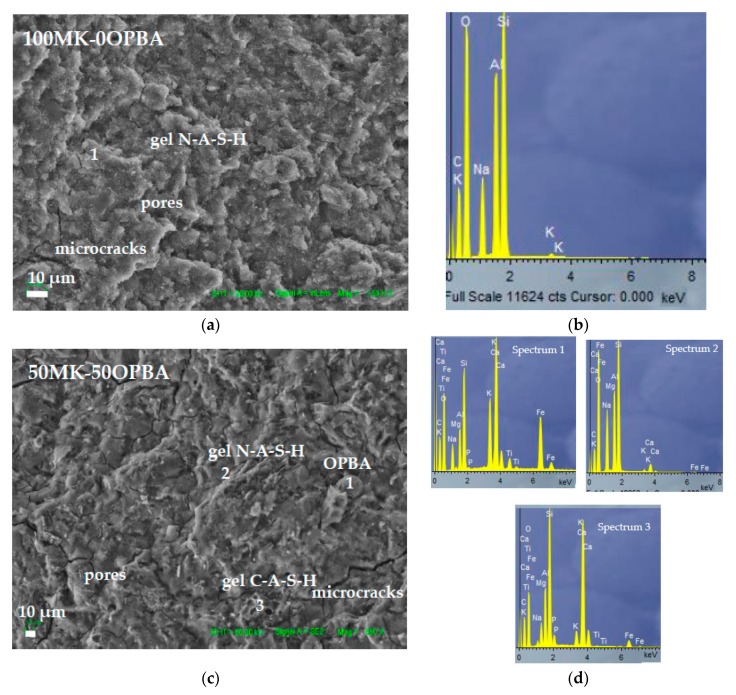

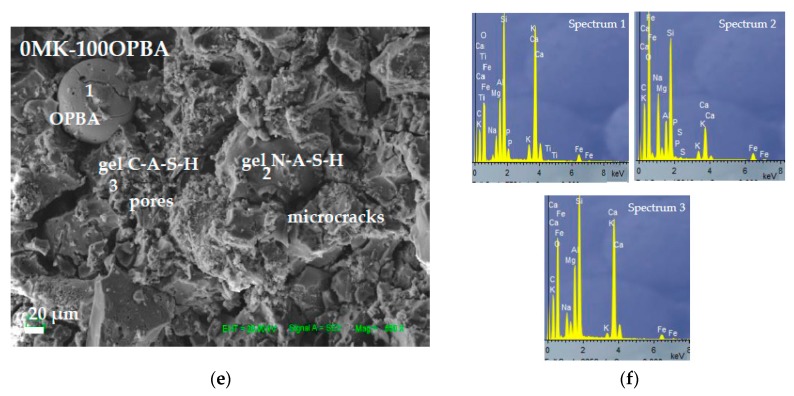
SEM micrographs and EDS analysis of (**a**,**b**) 100MK-0OPBA, (**c**,**d**) 50MK-50OPBA and (**e**,**f**) 0MK-100OPBA geopolymers after 28 days of curing.

The microstructure of the specimens is shown in Figure 10 through the SEM micrographs of geopolymers after 28 days of curing. The formation of the geopolymeric gel in all samples can be observed. In the 100MK-0OPBA control geopolymers, (Si/Al = 1.6), the sodium aluminosilicate hydrate (N-A-S-H) gel is observed by EDS. The presence of calcium in the biomass bottom ashes promotes the formation of other types of products, mainly aluminum-modified calcium silicate hydrate (C-A-S-H) gel, with a different kinetics and reaction degree to the N-A-S-H geopolymer gel [31]. The EDS analysis of the 50MK-50OPBA (Si/Al = 2.3) and 0MK-100OPBA geopolymers (Si/Al = 4.6) indicates the coexistence of the two C-A-S-H and N-A-S-H gels, the latter containing small amounts of calcium (N, C)-A-S-H. Table 3 shows the results of the point analysis (EDS) on N-A-S-H gel and on the (N,C)A-S-H gel areas for the determination of Si / Na and Si / Ca ratios. The results obtained show that N-A-S-H gels have different Si / Na ratios, increasing this ratio as increasing amounts of OPBA waste are incorporated. In C-A-S-H gels, a decrease in the Si / Ca ratio is observed as increasing amounts of waste are added.

This fact is due to the significant amount of calcium present in the OPBA waste. The appearance of the gel (N,C)-A-S-H is later than that of N-A-S-H, because calcium ions, together with those of aluminum, diffuse through the matrix formed. Thus, a small number of calcium ions interact with the N-A-S-H gel to form the gel (N,C)-A-S-H. The appearance of these two gels is indicative of the formation of the geopolymer [26]. These products, in general, have a positive effect on the mechanical strength of the material [32,33]. The geopolymers have a denser structure as larger amounts of biomass bottom ash are added, according to the bulk density data. Spherical voids, due to air bubbles during the geopolymerization process can be observed. Also, fracture microcracks generated both by the shrinkage of the material and water evaporation, which is characteristic in geopolymer systems with high SiO_2_/Al_2_O_3_ ratios [34], are also visible. The microcracks are larger in the geopolymer sample that uses only OPBA as raw material (0MK-100OPBA) due to the high molar ratio Si/Al = 4.6 of the specimens. In the samples incorporating OPBA waste as raw material, the presence of OPBA particles that have not reacted and that are embedded in the gel of the products formed can be observed. These particles of OPBA exhibit a spherical shape with the growth of some reaction products on the surface or partial dissolution of the outer layer [34]. The 0MK-100OPBA geopolymers present a greater amount of unreacted residue, according to the degree of reaction (Table 1), resulting in a smaller amount of geopolymeric gel formed.

#### 3.2.5. Compressive strength of MK-OPBA Geopolymers

**Figure 11:**
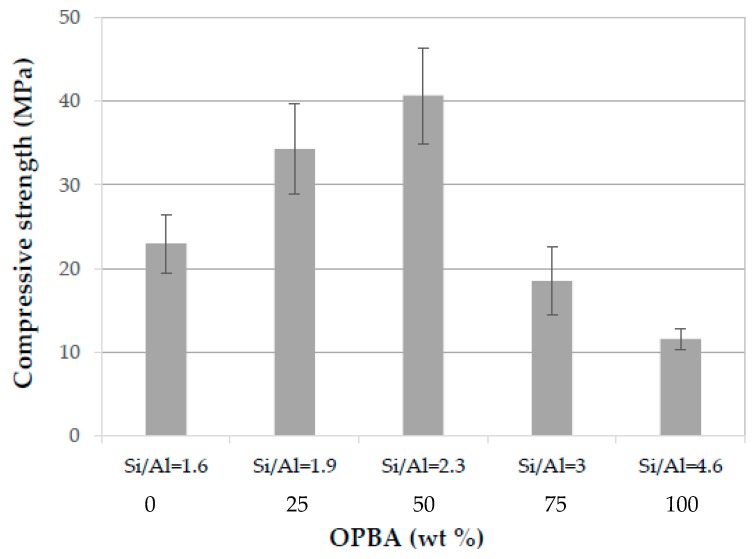
Compressive strength of the MK-OPBA geopolymers after 28 days of curing as function of the OPBA waste wt. % content and the Si/Al molar ratio.

The compressive strength of geopolymers is shown in Figure 11. The compressive strength of the control geopolymers 100MK-0OPBA, with a Si/Al molar ratio of 1.6, is 23 MPa. The incorporation of up to 50 wt.% of OPBA waste produced an increase in compressive strength, obtaining a maximum value of 40.1 MPa for the 50MK-50OPBA geopolymers (Si/Al = 2.3). The incorporation of OPBA waste, which contains calcium, results in the formation of the geopolymeric CASH gel, in addition to the NASH gel, as indicated by SEM micrographs and EDS analysis, increasing the compressive strength of the geopolymers. Therefore, the increase in mechanical strength in geopolymers containing up to 50 wt.% of OPBA can be attributed to the coexistence of N-A-S-H gel obtained after MK activation, together with C-N-A-S-H gels formed after activation OPBA waste [35,36]. Previous studies indicate that the optimum compressive strength is obtained for metakaolin-based geopolymers with Si / Al molar ratios between 1.8 and 2.2 [37,38,39]. The addition of 75 wt.% and 100 wt.% of bottom ash biomass, 25MK-75OPBA and 0MK-100OPBA geopolymers, with a Si/Al molar ratio of 3.0 and 4.6, respectively, despite presenting a more compact structure, with higher bulk density and lower porosity, due to the greater incorporation of the source of calcium, have lower values of compressive strength, 18.5 and 11.6 MPa respectively. This decrease in mechanical properties may be attributed to the larger size of the visible microcracks, probably as a result of drying shrinkage and removing structural water that propagate through the matrix easily, as well as the smaller amount of geopolymer gel formed as indicated degree of reaction and the greater amount of unreacted ashes, which leads to geopolymers with less mechanical strength due to these defects in the microstructure as indicated SEM micrograph and XRD and FTIR data.

#### 3.2.6. Thermal Conductivity of MK-OPBA Geopolymers

**Figure 12:**
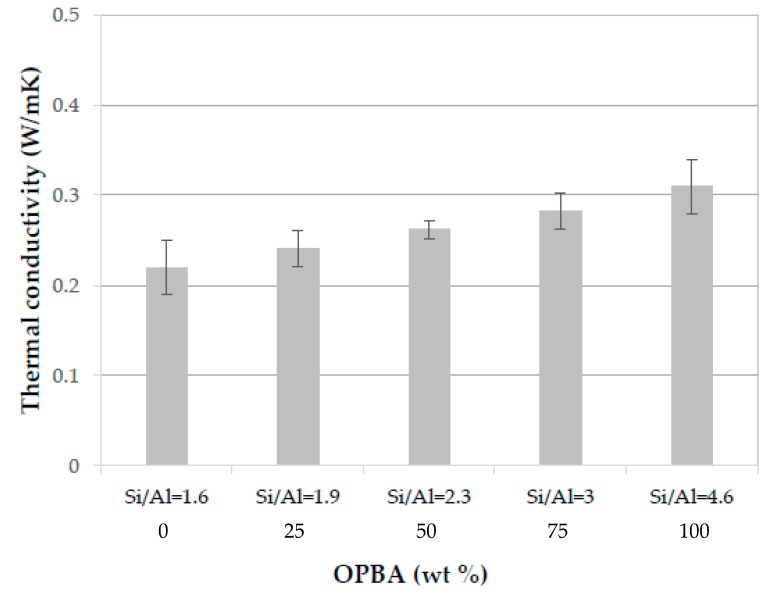
Thermal conductivity of the MK-OPBA geopolymers after 28 days of curing as function of the OPBA waste wt. % content and the Si/Al molar ratio.

The thermal conductivity data at 10 °C of the geopolymers after 28 days of curing can be seen in Figure 12. The thermal conductivity of the control geopolymers (100MK-0OPBA) with a Si/Al molar ratio of 1.6 is 0.22 W/mK, increasing as higher amounts of OPBA waste are substituted by MK, up to 0.31 W/mK for geopolymers containing as raw material only biomass bottom ashes (0MK-100OPBA specimens) (Si/Al = 4.6). These results are in agreement with the bulk density data, with the denser geopolymers with a lower thermal insulation capacity. However, the 50MK-50OPBA geopolymers, with a Si/Al molar ratio of 2.3, have a thermal conductivity of 0.26 W/mK, which represents a slight increase of this property with respect to the control geopolymers. The increase in thermal conductivity of geopolymers based on MK with the increase in the Si/Al molar ratio has been reported by other authors. Kanseu et al, 2011 [40] synthetized MK geopolymers using a mix of sodium and potassium hydroxide (~7.5 M) as well as sodium silicate as activator and established thermal conductivity values that increase linearly from 0.28 W/m K for a Si/Al = 1.3 to 0.35 W / m K for a molar ratio Si/Al = 2.5. Other authors have reported thermal conductivity values similar to those obtained in this study. Villaquirán-Caicedo et al. [41] obtained geopolymers based on metakaolin (MK) and alternative silica-based activators. The activators were produced by mixing rice husk ash (RHA) and silica fume (SF) with potassium hydroxide (KOH). The values of thermal conductivity of the geopolymers were between 0.17–0.35 W/mK. Thermal conductivity values of up to 0.31 W/m K have been obtained for metakaolin-based geopolymers with a Si/Al ratio of 1.9 using potassium silicate [42] and an Si/Al ratio of 1.8 using sodium silicate [43]. Geopolymers synthesized using as raw materials MK and OPBA waste under the established synthesis conditions have low thermal conductivity.

## 4. Conclusions

The results obtained in this work reveal the potential use of OPBA with high calcium content (19.7 wt.%) as a precursor in the manufacture of metakaolin-based geopolymeric materials. Alkaline activation of precursors with Si/Al and Na/Si molar ratios between 1.63–4.63 and 0.42–0.52 ranks, respectively, were obtained. The OPBA raw material allowed the obtaining of materials with cementing characteristics that can acquire maximum compressive strength of up to ~ 40 MPa with 28 days of curing (50MK-50OPBA geopolymers). The incorporation of calcium-rich ashes resulted in a denser and more compact structure, as well as the formation of other types of cementitious products, which contributes to the increase in mechanical performance until the incorporation of up 50 wt.% of OPBA waste. The addition of greater OPBA incorporations (75–100 wt.%) results in geopolymers with worse mechanical properties, possibly due to the lower amount of geopolymeric gel, and the higher amount of unreacted ash and the formation of larger microcracks in the matrix. The thermal conductivity increases with the replacement of increasing amounts of OPBA waste by MK precursor according to the bulk density data. However, all synthetized geopolymers have low values of thermal conductivity, between 0.22 and 0.31 W/mK. The geopolymers developed in this work show adequate mechanical properties. The incorporation of 50 wt.% of OPBA waste results in geopolymers with better mechanical properties. These new construction materials can be used as substitutes for materials whose environmental cost is much higher when they are produced, such as Portland cement.

## Figures and Tables

**Table 1:** Quantities of raw materials and, Si/Al, Si/Na molar ratio and Degree of reaction of synthesized geopolymers.

Sample	Si/Al Molar Ratio	Si/Na Molar Ratio	MK (g)	OPBA (g)	NaOH (g)	Water (g)	Na_2_SiO_3_ (g)	Liquid/Solid Ratio	Degree of Reaction (%)
100MK-0OPBBA	1.63	0.42	450.0	0	80	195	300	0.85	54
75MK-25OPBA	1.91	0.44	337.5	112.5	80	195	300	0.85	50
50MK-50OPBA	2.32	0.46	225.0	225.0	80	195	300	0.85	48
25MK-75OPBA	3.05	0.49	112.5	337.5	80	195	300	0.85	42
0MK-100OPBA	4.62	0.52	0	450.0	80	195	300	0.85	33

**Table 2:** Chemical composition of olive-pine bottom ash (OPBA) and metakaolin (MK) raw materials.

Oxide Content (%)	OPBA	MK
**SiO_2_**	46.10	58.03
**Al_2_O_3_**	12.04	40.29
**Fe_2_O_3_**	4.78	0.42
**CaO**	19.65	0.09
**MgO**	3.71	0.11
**MnO**	0.09	0.01
**Na_2_O**	0.78	0.02
**K_2_O**	4.59	0.39
**TiO_2_**	0.83	0.15
**P_2_O_5_**	1.12	0.07
**SO_3_**	0.41	0.01
**LOI**	5.58	0.36

**Table 3:** Determination of the atomic relationship by EDS.

Sample	Si	Al	Na	Ca	Si/Al	Si/Na	Si/Ca
100MK-0OPBA Gel NASH	27.92	15.40	8.08	0	1.81	3.46	-
50MK-50OPBA Gel NASH	23.63	12.45	12.50	4.56	1.90	1.89	5.18
50MK-50OPBA Gel CASH	17.77	7.86	4.25	23.70	2.26	4.18	0.75
0MK-100OPBA Gel NASH	25.40	12.55	12.70	2.40	2.03	2.0	10.60
0MK-100OPBA Gel CASH	16.38	6.14	2.80	25.25	2.66	5.84	0.64
